# Impact of total parenteral nutrition standardization led by pharmacist on quality in postoperative patients with colorectal cancer

**DOI:** 10.1038/s41430-018-0281-0

**Published:** 2018-10-17

**Authors:** Zeng Wang, Yunsong Peng, Xinjun Cai, Yingying Cao, Guonong Yang, Ping Huang

**Affiliations:** 1 Department of Pharmacy, Zhejiang Cancer Hospital, 310022 Hangzhou, P.R. China; 2 Department of Pharmacy, Hangzhou Red Cross Hospital, 310003 Hangzhou, P.R. China

**Keywords:** Nutrition, Translational research

## Abstract

**Background/objectives:**

Abdominal surgery significantly affects the structure and function of the gastrointestinal system of patients, total parenteral nutrition (TPN) is an important nutrition support method for postoperative patients. However, in the process of TPN practice, the excessive fat emulsion and compound amino-acid prescriptions ratio are often prescribed by doctors. To address the problem, we developed the computerized TPN prescription management system to promote the personalized provision of TPN. The purpose of this study is to evaluate the intervention effects of the computerized TPN prescription management system, which is designed by pharmacists in the Surgical Department of Abdominal Oncology at Zhejiang Cancer Hospital in July 2015.

**Subjects/methods:**

The computerized TPN prescription management system applied in Surgical Department of Abdominal Oncology on 1 July 2015. The computerized TPN prescription management system was evaluated by comparing the patients who were treated 3 months after the application of the system with the control subjects who were treated 3 months prior to the application of TPN prescription management system in Surgical Department of Abdominal Oncology.

**Results:**

In total, 218 TPN prescription-treated patients with colorectal cancer received surgery treatment were analyzed, including 121 subjects who received the treatment 3 months prior to application of TPN prescription system (IPN period) and 97 subjects who received the treatment after 3 months of the system application (SPN period). The rates of optimized TPN prescriptions are 47.1% and 88.7% prior to and after application of TPN prescription review system, respectively (*p* < 0.001). In detail, prior to application of TPN prescription review system, abnormal glucose–lipid ratio and nitrogen–calorie ratio are the most common problems, which accounted for 74.3 and 97.9%, respectively (*p* < 0.01). Whereas the proportion of the insufficient dosage of amino acids is 62 and 96.9%, respectively (*p* < 0.01). Other problems are insufficient dosage of insulin and excessive fat soluble vitamin supplement. After application of TPN prescription review system, as the glucose–lipid ratio and nitrogen–calorie ratio are set up in fixed range according to the nutrition treatment guidelines, only a small amount of TPN prescriptions have the problem of insufficient dosage of compound amino acid. Furthermore, before and after the application of TPN management software, the gender, age, performance status (PS) score and BMI index of the two groups of colorectal cancer patients were not statistically different (*p* > 0.05). There were significant differences in albumin and prealbumin between the two groups after operation (*p* < 0.05), and there was a significant difference in total protein (*p* < 0.001). There were significant differences in alanine aminotransferase and indirect bilirubin between liver and kidney function (*p* < 0.01), and there were significant differences in aspartate aminotransferase and total bilirubin (*p* < 0.05). Other total cholesterol, l-γ-glutamyl transferase, direct bilirubin and creatinine were not statistically different (*p* > 0.05). Blood routine (WBC, Hb and lymphocyte), length of stay and recurrence rate were not statistically different (*p* > 0.05).

**Conclusions:**

The application of TPN management software not only standardized the doctor’s TPN medical advice, but also improved the qualified rate of TPN doctor’s advice, thus ensuring the safety of the patient’s medication. It also had a positive effect on postoperative recovery of colorectal cancer patients, and ensured the efficacy of the treatment of patients. In addition, it reduced the workload of the pharmacist’s audit prescription and improved the efficiency of the audit prescription, and further emphasized the role and value of pharmacists.

## Introduction

A majority of the cancer patients exhibited different degrees of malnutrition and immune dysfunction before surgery, which might be attributed to the stress response to surgery and increased metabolism. Malnutrition also leads to immune suppression, accompanied by extreme weight loss [1]. In addition to other maladies, resulting in patients with postoperative infection, gastroparesis and other related complications, which seriously affect the patient’s physical recovery and quality of life [2, 3]. As malnutrition and nutritional imbalance are common and cannot be managed easily in patients with cancer, postoperative nutritional support for patients with malignant tumors is imperative.

Malnutrition is also an important cause of cancer progression and deaths [4]. Previous studies have shown severe malnutrition has a negative effect on immune function and the resistance to antitumor therapy; moreover, it is also associated with an increased incidence of postoperative complications. Thus, an appropriate reasonable nutritional support for malnourished patients with cancer is absolutely essential. Although parenteral nutrition has been greatly developed, the implementation process requires the doctors to adjust the nutritional substrate according to the patients’ condition. Prescriptions with inadequate knowledge of various nutrition and other factors involved leads to the increased adverse drug reactions. Systemic metabolic changes take places in cancer patients, for example, increased liver glycogen synthesis and tumor load is proportionally correlated with increased pancreatic beta-cell sensitivity to glucose drop in gastrointestinal tract cancer patients, this phenomenon is similar to patients with type 2 diabetes; leading to increased protein, amino-acid and lipid metabolism and abnormal protein, glycerin and fatty acid conversion rate [5, 6].

Total parenteral nutrition (TPN) refers to the nutritional support of all nutrients obtained completely outside the gut. However, excess glucose, lipids and calories from TPN would bring many complications, such as hepatobiliary alterations, hyperglycemia and hypertriglyceridemia. As high concentrations of glucose will cause a burden on blood sugar and cardiopulmonary function; excessive liver fat deposition can be caused, triggering fatty liver, liver damage and cholestasis.

TPN also need to provide patients with all kinds of essential amino acids and non-essential amino acids, is the only nitrogen source of TPN. If amino acids are given separately without glucose supply, energy supply for human body will be insufficient. Organizations can only convert exogenous amino acids into sugars through the energy pathway of the pancreas, and then amino acids will not be able to promote protein synthesis when consumed. Too fast or excessive infusion of amino acids will cause damage to the brain and liver function. How to regulate its rational use, to ensure the safety of patients and reduce unnecessary medical expenses, are a problem for hospital administrators.

Research results show that the use of information automation tools to review prescriptions and medical records can reduce medication errors a lot [7]. Therefore, in order to solve the problem of insufficient management of physician unreasonable TPN medical prescriptions, the research group developed TPN medical management software, in the early stage when relative indexes are calculated, with the pre-set range to control the rationality of the TPN doctor’s advice, pointing out the unreasonable part of the TPN group. However, the impact of this software intervention on postoperative recovery and prognosis of cancer patients is unknown.

In view of this, we collected 218 patients with colorectal cancer surgery in our hospital to evaluate the effect of TPN standardization led by pharmacist.

## Methods

### The development of TPN control software

In the Windows platform, under the Chinese operating system environment, based on the hospital information system (HIS, EMR electronic medical records), tumor specialist hospital pharmacist-led TPN control software isdeveloped by using C# program editing tools and ORACLE 11G database system. When the software is developed, the pharmacist sets the corresponding formula for each prescription index, and the doctor’s TPN medical order data will be automatically converted into information data, which falls into the column of index value. In addition, the range of total calories, glycolipid ratio, nitrogen–calorie ratio, electrolyte concentration and insulin usage were used as reference values. If you do not meet the requirements or there is a lack of drug in the TPN orders, in the interface below an error message will appear, and the TPN order will not be billed and saved until the indicators fully meet the requirements.

### Patients information

In July 2015, a standard TPN regime in adult patients was introduced in our hospital for routine use. Following implementation of this new procedure, the effects were assessed by retrospective analysis. Individualized TPN episodes in adult patients between April 2015 and June 2015 (IPN period) were compared with a similar group of patients who had received parenteral nutrition (PN) formulations between September 2015 and December 2015 (SPN period). The study was approved by the Medical Ethics Review Committee of Zhejiang Cancer Hospital (Hangzhou, China).

#### The object of study

According to the inclusion criteria, the clinical data of 218 cases of colorectal cancerpatients after surgery were collected. Of which, TPN control software was employed in the TPN prescription management of 97 cases and TPN prescription of other 121 cases were not managed by the TPN control software.

#### Inclusion standard

(1) Colorectal cancer; (2) the pathological diagnosis was malignant; (3) surgical patients (laparoscopic or local resection surgery or colonoscopy local excision or exploration operation except); (4) hospitalization time > 7 days, TPN treatment after the operation was applied for at least 7 days; (5) aged 18–75 years; (6) the liver, kidney and bone marrow function meet the requirements Aspartate transaminase (AST) < 2 times the upper limit of normal, white blood cell count ( > 3.5 × 109/L), platelet count ( > 100 × 109/L), serum creatinine was 1.5 times the upper limit of the normal value). The patients who do not meet any of the inclusion standard should be removed.

#### Research methods

##### Electronic medical record information collection

Method of retrospective investigation was used to collect the basic clinical information and TPN use-related information, including age, gender, height, weight, basic disease, TPN number of days, specific drugs, hospitalization time, blood routine, liver function test results and so on via the electronic medical record system.

##### Energetic and amino-acid requirements for adults per day

The standard energetic and amino-acid requirements applied in this study for adults per day are as follows:

Glucose 2–4 g/(kg day);

Lipid 1.2–1.5 g/(kg day);

Amino acid 0.8–2 g/(kg day).

##### Follow-up

Telephone follow-up was performed on postoperative recurrence of colorectal cancer.

### Statistical analysis

The SPSS software version 20.0 (Chicago, IL, USA) was used for all analyses. Student’s *t*-test was used to compare mean age between cases and controls. Other data were assessed by the chi-square test, with *p* < 0.05 considered statistically significant.

Variable definition

total calories: glucose and fat calories

glycolipid ratio: glucose calorie/fat calorific

nitrogen–calorie ratio: glucose and fat calories/nitrogen content.

## Results

### Basic information of patients

As can be seen from Table 1, no significant difference was found in sex, age, disease type, PS score and body mass index (BMI) index between the group in Individualinzed parenteral nutrition (IPN) period and the group in Standardized parenteral nutrition (SPN) period (*p* > 0.05).

**Table 1:** Basic information of colorectal cancer patients using TPN

	Sort	IPN period (*n*=121)		SPN period (*n*=97)	
		Number	Proportion (%)	Number	Proportion (%)
Gender	Male	78	64.5	63	64.9
	Female	43	35.5	34	35.1
Age (y)	> 65	44	36.4	31	32.0
	≦65	77	65.6	66	68.0
BMI (kg/m^2^)	<18.5	14	11.6	7	7.2
	18.5–25	77	63.5	67	69.1
	> 25	30	24.9	23	23.7
Type of malignant tumor	Colorectal malignant tumor	67	55.4	68	70.1
	Colonic malignant tumor	54	44.6	29	29.9

### TPN medical advice pass rate

The effect of TPN management software on the rationality of TPN orders in colorectal cancer surgery patients is specifically shown in Table 2. Before the software application, the unreasonable lipid ratio and thermal nitrogen ratio, respectively, accounted for 25.7and 38%, and in the use of software in 97 cases, both unreasonable accounted for only 2.1 and 3.1%, with significant difference (*p* < 0.01). At the same time, the rationality of the use of alanyl glutamine increased from 90.9 to 100%, with statistical difference (*p* < 0.05). From the rationality of insulin use, there was no statistical difference between the group in IPN period and the group in SPN period. TPN total prescription qualified rate increased from 47.1 to 88.7%, *p-*value <0.001, there was extremely significant statistical difference. Generally speaking, the use of the TPN software significantly improved the rationality of the prescription.

**Table 2:** Case of TPN composition suitability

TPN ingredient	Section	IPN period		SPN period	
		Number	Proportion (%)	Number	Proportion (%)
Glucose/fat	<1	31	25.7	2	2.1
	1–3	90	74.3	95	97.9
	>3	0	0.0	0	0.0
Total energy/N	<100	35	28.9	3	3.1
	100–200	75	62.0	94	96.9
	> 200	11	9.1	0	0.0
Rationality of using propylene ammonia amide (<0.4)	Yes	110	90.9	97	100.0
	No	11	9.1	0	0
Rationality of using insulin	Yes	120	99.2	96	99.0
	No	1	0.8	1	1.0

### Improvement of nutrition index

The results from Table 3 showed that before TPN treatment, the plasma protein level in the two groups of patients did not differ significantly, while after TPN treatment, the albumin, prealbumin and the total protein content of the TPN-treated patients in SPN group were higher than that of IPN group, and all three proteins had significant difference (*p* < 0.01).

**Table 3:** Nutritive index results

	IPN period		SPN period	
	Before TPN treatment	After TPN treatment	Before TPN treatment	After TPN treatment
Albumin	33.69 ± 6.64	35.77 ± 5.75	34.28 ± 5.96	37.70 ± 3.35**
Prealbumin	159.00 ± 57.89	134.48 ± 48.01	160.98 ± 51.95	163.69 ± 39.93**
Total protein	55.69 ± 9.29	60.18 ± 7.09	56.71 ± 7.99	64.08 ± 5.01**

Values are expressed as mean ± SD, or %

Compared with IPN group after TPN treatment *p* < 0.05; **p < 0.01

### Influence of liver, kidney function and routine blood test

Table 4 shows that after using the software, alanine and indirect bilirubin was significantly lower than before (*p* < 0.01), aspartic acid and total bilirubin is less than before (*p* < 0.05). However, there was no significant difference in blood routine between the two groups (*p* > 0.05).

**Table 4:** Hepatic function and renal function results and blood routine examination results

	IPN period	SPN period
Total cholesterol	3.37 ± 0.48	3.50 ± 0.49
Alanine	21.18 ± 5.92	14.08 ± 5.76**
Aspartic acid	21.10 ± 5.64	18.46 ± 5.98*
l-gamma glutamine transferase	49.09 ± 27.43	41.61 ± 24.67
Total bilirubin	12.42 ± 2.22	10.87 ± 4.24*
Direct bilirubin	5.14 ± 2.04	4.68 ± 3.19
Indirect bilirubin	7.33 ± 1.69	6.22 ± 1.70**
Creatinine	59.71 ± 10.51	59.03 ± 13.93
White blood cell	7.53 ± 1.98	7.66 ± 2.04
Hemoglobin	11.75 ± 1.50	11.33 ± 2.38
Lymphocyte	1.08 ± 0.30	1.03 ± 0.37

Values are expressed as mean ± SD, or %

*p < 0.05; **p < 0.01

### Postoperative patient status

The discharge weight of IPN group and SPN group were 58.95 ± 11.14 kg and 59.50 ± 9.47 kg, length of postoperative hospital stay of IPN group and SPN group were 12.91 ± 7.35 days and 15.06 ± 9.72 days. There was no significant difference in discharge weight and length of hospital stay between the two groups (*p* > 0.05). Moreover, the chemotherapy rate of IPN group and SPN group were 28.1 and 37.1% (*p* > 0.05), and the recurrence rate of IPN group and SPN group were 5 and 4.1% (*p* > 0.05).

## Discussion

After surgery, colorectal cancer patients exhibited enhanced decomposition of the body. This phenomenon was often accompanied by immune function and protein loss. A reasonable ratio of TPN treatment can provide high quality and effective nutritional support to patients for improving postoperative recovery and the success rate of surgery [8, 9]. In our study, it suggests that the use of TPN prescription management system led by pharmacist not only reduced the workload of clinical pharmacists, but also improved the rationality of TPN prescription, thereby improving rapid recovery of nutritional indicators of patients.

In order to ensure the safe use of drugs by patients, the pre-review prescription is very necessary. TPN prescriptions are different from general medicines, in that they contain a variety of drugs and are mainly formulated by mixing preparations such as glucose, fat emulsions, amino acids, vitamins, electrolytes and trace elements. Moreover, it is generally necessary to evaluate the rationality of the prescription by using the glycolipid ratio, nitrogen–calorie ratio, the amount of amino-acid used and the amount of insulin used. The pharmacist’s manual auditor needs to calculate so much data that it is obviously time consuming and laborious, and it cannot guarantee accuracy. The development of TPN prescription auxiliary review procedures have been already reported by domestic and foreign scholars. Ouyang et al. [10] use Excel to set the appropriate auditing indicators and calculation formulas so as to shorten the pharmacist manual review time. Lehmann et al. [11] designed an on-line TPN calculation system for use in neonatology, thereby reducing the TPN doctor’s error rate. However, these TPN prescription auxiliary review procedures cannot be embedded in the hospital HIS system and connect with the actual clinical information of patients, nor can it intercept unreasonable prescriptions.

Therefore, in order to solve the above deficiencies, our hospital pharmacists developed the computerized TPN prescription management system with valuable clinical application. With the help of information management and control measures, the problem of calculation of each parameter can be solved, moreover, unreasonable prescriptions would be alerted and intercepted according to the range of control values, thus, safeguarding the rationality of prescriptions from the source.

In this study, a total of 218 patients with TPN were analyzed, including 121 cases before application software and 97 cases after the application of the software. Consequently, TPN prescription optimization rate was 87.7%. In detail, prior to the application of TPN prescription review system, abnormal glucose–lipid ratio and nitrogen–calorie ratio were the most common problems, accounted for 25.7% and 38.0%. The three most important components of TPN are glucose, fat emulsion and amino acids. Glucose and fat milk are the main sources of energy, which are called non-protein calories and account for 85% of the body’s required energy [12]. According to clinical nutrition guidelines, the glycolipid ratio should be within the range of 1.0–3.0. The glycolipid ratio is too high or too low, suggesting that the patient’s glucose or fat supply ratio is too large. Furthermore, the reasonableness of the nitrogen–calorie ratio largely influences the utilization of amino acids for the synthesis of proteins. When the hot-to-nitrogen ratio is 100 to 200 cal/g, the normal nitrogen balance occurs. The nitrogen–calorie ratio is too high, suggesting that the patient’s amino-acid supplement is insufficient, and the tissue protein in the body is still decomposing, and in the long term, negative nitrogen balance is likely to occur. The nitrogen–calorie ratio is too low, which means that the human body uses amino acids as the main energy-supplying substance, causing waste of amino-acid supplementary. The application of TPN control software sets the glycolipid ratio and the nitrogen–calorie ratio within the normal range according to nutritional treatment guidelines, only a very small amount of TPN prescription presents the above problems.

Moreover, whether or not the rationality of the TPN prescription directly affects the recovery of the patient’s nutritional level, this study continues to analyze the changes in the expression level of related proteins before and after application of TPN control software. As plasma protein levels are a common indicator of the nutritional status of the body’s proteins, which not only provide objective nutritional evaluation results, but are not susceptible to subjective factors. The most commonly used indicators include serum albumin, prealbumin and total protein. Albumin and prealbumin are synthesized by the liver protein, when the liver is damaged when the synthesis is reduced, the blood content is also reduced accordingly. Studies have shown that serum albumin levels are one of the reliable predictors of malnutrition [13–15], and this phenomenon is closely related to clinical outcomes and are widely used as a tool for predicting morbidity and mortality [16]. However, due to albumin sensitivity is poor, prealbumin is commonly measured, which exhibits a primary function of transporting thyroxine and vitamin A. Also, it has thymus hormone activity, which can promote lymphocyte maturation to enhance the body immunity [17]. Its half-life is short, only about 12 h–1.9 days, and the content is less only 0.4% of total plasma protein content. Serum prealbumin indicator can faster reflect the function of liver synthesis and secretion of protein, and acute changes in the nutritional status and early metabolism levels in the body [18]. This study showed that albumin, prealbumin and total protein in the patient group were significantly higher and significantly different compared with those before software application. The results suggest that the use of the control software is more conducive to the recovery of patients with colorectal cancer after surgery.

In the process of clinical application, the timing of TPN selection, the ratio of nutritional components and the combination of drugs are essential, otherwise adverse reactions might occur. The adverse effects of TPN are largely due to unscientific proportions of nutrients, such as glycolipid ratio, nitrogen–calorie ratio and the ratio of alanyl glutamine to amino acids, which are not within the specified limits. In this study, we also evaluated the changes of hepatorenal and renal function before and after the application of TPN management software. The results showed that the control results were better than before, but the related indicators did not exceed the normal upper limit.

However, there were no statistical difference between the two groups in terms of length of postoperative hospital stay, discharge weight and chemotherapy rate, which was probably due to the small sample size. The postoperative recurrence rate was not statistically different, which were related to the short follow-up periods. This study will continue to follow 5-year and 10-year recurrences.

To sum up, the application of the TPN control software which guided by the pharmacist, significantly improved the rationality of the use of TPN drugs. It is also beneficial to improve the postoperative nutritional indicators of patients. In addition, it reduced the workload of pharmacists, improved the efficiency of prescription review, thereby suggesting the role and value of pharmacists.

## Electronic supplementary material

supplementary information
