# Supplementary material for: Impact of total parenteral nutrition standardization led by pharmacist on quality in postoperative patients with colorectal cancer
Source: Eur J Clin Nutr. 2018 Oct 17;73(2):243–9. doi: 10.1038/s41430-018-0281-0 (PMC6368531; doi:10.1038/s41430-018-0281-0)
Supplement: Supplementary file 1 — supplementary information [file 41430_2018_281_MOESM1_ESM.docx]

supplementary information

**Figure legends**

Figure 1. TPN control software was issued to remind the clinician (Chinese)

When the software is developed, the pharmacist sets the corresponding formula for each prescription index, and the doctor's TPN medical order data will be automatically converted into information data, which falls into the column of index value, including the range of total calories, glycolipid ratio, heat/nitrogen ratio, electrolyte concentration and insulin usage and so on.

Tables

Table 1. TPN control software was issued to remind the clinician

| Drug name/specification | dose | Quantity | Indicator name | Index value | Reference  Value |
| --- | --- | --- | --- | --- | --- |
| Specific error message tips | | | | | |

Table 2 Postoperative relapse status

|  |  | control group（n=121） | | Intervention group（n=97） | |
| --- | --- | --- | --- | --- | --- |
|  |  | quantity | ratio% | quantity | ratio% |
| relapse | yes | 6 | 5.0 | 4 | 4.1 |
|  | no | 115 | 95 | 93 | 95.9 |
| Chemotherapy | yes | 34 | 28.1 | 36 | 37.1 |
|  | no | 87 | 71.9 | 61 | 62.9 |
